# Prevention effect of rare ginsenosides against stress-hormone induced MTOC amplification

**DOI:** 10.18632/oncotarget.9059

**Published:** 2016-04-27

**Authors:** Jung-Hyun Cho, Ho-Young Chun, Jung Suk Lee, Jee-Hyun Lee, Kyu Jin Cheong, Youn-Sang Jung, Tae-Gyun Woo, Min-Ho Yoon, Ah-Young Oh, So-Mi Kang, Chunghui Lee, Hokeun Sun, Jihwan Hwang, Gyu-Yong Song, Bum-Joon Park

**Affiliations:** ^1^ Department of Molecular Biology, College of Natural Science, Pusan National University, Busan, Korea; ^2^ College of Pharmacy, Chungnam National University, Daejoen, Korea; ^3^ Department of Statistics, College of Natural Science, Pusan National University, Busan, Korea; ^4^ Department of Microbiology, College of Natural Science, Pusan National University, Busan, Korea

**Keywords:** stress hormone, MTOC, cancer prevention

## Abstract

Stress has been suggested as one of important cause of human cancer without molecular biological evidence. Thus, we test the effect of stress-related hormones on cell viability and mitotic fidelity. Similarly to estrogen, stress hormone cortisol and its relative cortisone increase microtubule organizing center (MTOC) number through elevated expression of γ-tubulin and provide the Taxol resistance to human cancer cell lines. However, these effects are achieved by glucocorticoid hormone receptor (GR) but not by estrogen receptor (ER). Since ginsenosides possess steroid-like structure, we hypothesized that it would block the stress or estrogen-induced MTOC amplification and Taxol resistance. Among tested chemicals, rare ginsenoside, CSH1 (Rg6) shows obvious effect on inhibition of MTOC amplification, γ-tubulin induction and Taxol resistance. Comparing to Fulvestant (FST), ER-α specific inhibitor, this chemical can block the cortisol/cortisone-induced MTOC deregulation as well as ER-α signaling. Our results suggest that stress hormone induced tumorigenesis would be achieved by MTOC amplification, and CSH1 would be useful for prevention of stress-hormone or steroid hormone-induced chromosomal instability.

## INTRODUCTION

Differentially from commonly accepted hypothesis, it has not been clearly demonstrated that psychological or physiological stresses are really related with cancer initiation or progression [1–4]. Thus, detail molecular mechanism of stress-related human cancer has not been demonstrated. When human body is fronted to stressful condition, stress-hormones such as cortisol and ephinephrine are released from adrenal gland in response to pituitary gland-secreted Adrenocorticotropic hormone (ACTH) [5]. Indeed, increased level of cortisol produces several harmful reaction in human body such as depression, decrease of immune system, etc [6, 7]. However, these responses are not directly linked to cancer initiation.

Renal cell carcinoma (RCC) is human malignancy in kidney, which is adjacent with adrenal gland. In addition, although several reasons have been suggested such as aging, smoking, and others, stress is also pointed as reason for RCC [8–10]. In fact, serum cortisol level is increased in RCC patients [11, 12]. Although concerning this, suppressive effect of stress hormone on immune system has been generally suggested, it would be not sufficient for explanation of stress-induced cancer. Indeed, several epidemiological analysis indicate that stress can increase the incidence of various kinds of human cancers including colon and lung cancer [13, 14].

In previous, it has reported that under von Hippel-Lindau protein (VHL)-deficient condition, elevated estrogen receptor (ER)-α or Estrogen treatment provides Taxol resistance via amplification of MTOC [19]. Since MTOC is critical for maintaining of chromosome stability, deregulation of MTOC is linked to M-phase collapse or cancer aneuploidy [15–18]. In fact, VHL directly associates to ER-α and inhibits MTOC amplification. Under VHL-deficient condition, elevated ER-α disrupts BRCA1-Rad51 binding that suppresses MTOC amplification. Thus, suppression of ER-α by ER-α inhibitors such as tamoxifen or Faslodex, can restore the centrosome number in VHL-deficient RCC cell lines [19]. Since stress hormones also originated from steroid and share common chemical structure [20], it is very reasonable hypothesis that stress hormones also promotes MTOC amplification and Taxol resistance.

*Panax Ginseng* has been used for a long time to treat various kinds of human diseases including cancer and kidney malfunction [21, 22]. Recently, isolated ginsenoisdes has been reported to be effective on immune system [23]. However, molecular biological working mechanism of ginsenosides on human cancer has not been revealed until now. Thus, it would be meaningful to verify the working mechanism and the effect of ginsenosides on human cancer.

This study is focused on tumorigenic effect of stress hormone, in particular MTOC amplification and drug resistance. In addition, since several ginsenosides possess stress hormone-related chemical structure, favorable effect of ginsenoisde on MTOC amplification and Taxol resistance is investigated.

## RESULT

### Stress hormones provide taxol resistance

**Figure 1:**
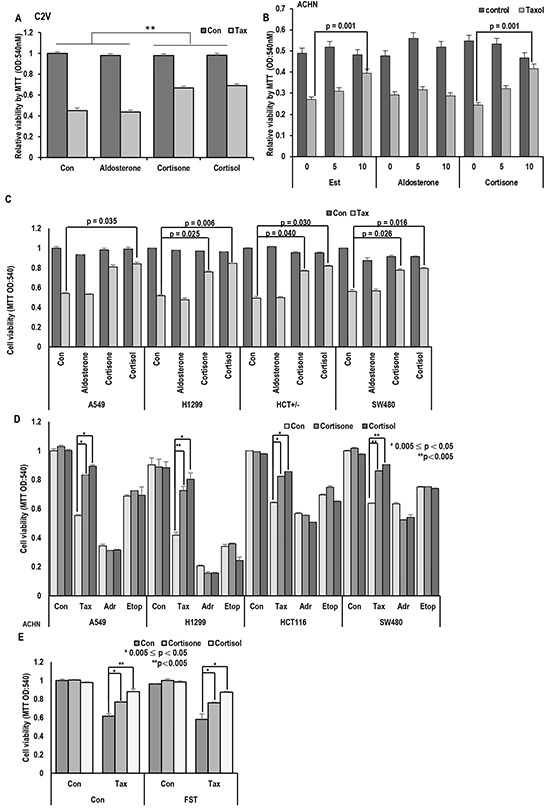
Stress hormone induces Taxol resistance **A.** Cortisone and Cortisol but not aldosterone block the Taxol-induced cell death. Aldosterone (5 μM), cortisone (5 μM), cortisol (5 μM), and Taxol (3 Mm; Tax) were treated for 72 hr in VHL-positive C2V cells. Cell viability was measured by MTT assay. ** mean different group by ANOVA test (p<0.001). **B.** Cortisone provides the Taxol resistance, like as Est. ACHN cells were treated with indicating dose of steroid hormones for 72 hr. Cell viability was determined by MTT assay. **C.** Cortisone and Cortisol but not aldosterone block the Taxol-induced cell death in non-RCC cell lines (Lung cance cell lines; A549, H1299, Colon cancer cell lines; HCT116, SW480). Aldosterone (5 μM), cortisone (5 μM), cortisol (5 μM), and Taxol (3 μM) were treated for 72 hr in VHL-positive C2V cells. Cell viability was measured by MTT assay. **D.** The specific effect of cortisone on Taxol-induced cell death. Differentially from Taxol (Tax), cortisone did not provide the resistance to DNA damage reagent such as Adriamycin (Adr) and Etoposide (Etop). Cells were incubated with indicated chemicals (Taxol 3 μM; cortisone 5 μM; Adr 2 μg/ml; Etop 10 μM) for 72 hr later. Cell viability was determined by MTT assay. **E.** Inhibition of ER- α via Faslodex (FST) does not block GH-induced Taxol resistance. ACHN was incubated with FST (3 μM), Taxol (3 μM), cortisone (5 μM) and cortisol (5 μM) for 72 hr. Cell viability was measured by MTT assay.

Since stress hormone, cortisol and its related hormones (cortisone and aldosterone) are commonly originated from cholesterol and show similar chemical structure with estrogen (Supplementary Figure S1A), their biological effect on Taxol-induced cell death was tested. Similarly with estrogen (Est) [19], cortisone and cortisol but not aldosterone provided Taxol resistance in two kinds of RCC cell lines (Figure 1a and Supplementary Figure S1B). Their inhibitory effect showed the dose-dependency (Figure 1B). Stress hormones can affect various kinds of tissues and cells [13, 24, 25], we next checked the effect of glucocorticoid hormones in lung and colon cancer cell lines and obtained the similar result that cortisol and cortisone inhibited Taxol-induced cell death as dose-dependent manner (Figure 1C and Supplementary Figure S1C). In these cell lines, aldosterone did not alter the Taxol-sensitivity even in high dosage (Supplementary Figure S1C). Next, we checked the effect of cortisone and cortisol on other kinds of anti-cancer drugs. Similarly to Est [19], cortisol and cortisone did not alter the sensitivity to Adriamycin or etoposide (Figure 1D and Supplementary Figure S1D). To know that cortisol/cortisone-induced Taxol resistance is achieved by ER- α/Est signaling cascade [19], we treated an ER-α inhibitor, Fulvestrant (FST), and measured the Taxol-sensitivity. However, FST did not block the cortisone/cortisol—induced Taxol resistance (Figure 1E), indicate that these hormone's effect on Taxol-induced cell death would be exerted by ER-α independent pathway.

### Stress hormone promote MTOC amplification

**Figure 2:**
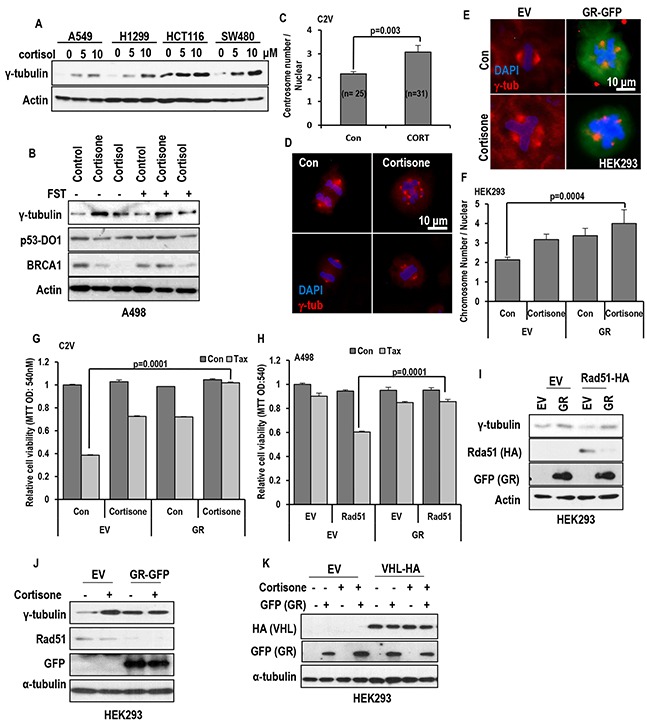
Stress hormone increases MTOC via GR **A.** cortisol obviously induced γ-tubulin in various cell lines. Each cells were treated with indicating dose of cortisol for 72 hr. Western blot was performed for measuring γ-tubulin expression. Actin was used for loading control. **B.** GHs induce γ-tubulin in regardless of FST. A498 (VHL deficient cell line) were incubated with cortisone (5 μM) and cortisol (5 μM) with/without FST (3 μM) for 24 hr. However, BRCA1 expression was increased by FST, although reduction of BRCA1 expression by GH was not affected. In addition, p53 expression was not altered by FST or GH. Actin was used for loading control. **C** and **D.** The number of MTOC is increased by cortisone treatment (CORT; 5 μM) in VHL-intact C2V cells. About 50 cells were counted in each conditions to check average MTOC numbers and representative pictures were shown. Cells were stained with anti-γ-tubulin antibody (Red) to detect MTOC and DAPI (Blue) for DNA. **E.** GR and cortisone can induce MTOC amplification. After GR transfection or cortisone (5 μM) treatment, HEK293 cells were stained with anti γ-tubulin antibody (Red) and DAPI (Blue). **F.** Addictive effect of GH and GR on MTOC amplification. About 50 numbers of cells were counted in each condition to estimate MTOC numbers. **G.** Overexpressed GR can provide the resistance to Taxol in VHL-positive C2V cells. GR was transfected to C2V cells and Taxol (Tax; 3 μM) was treated. 72 hr later, cell viability was measured by MTT assay. **H.** GR blocks Rad51-mediated Taxol-sensitization. Re-sensitization to Taxol by Rad51 overexpression in VHL-deficient A498 was erased by GR overexpression. A498 cells were transfected with GR and/or Rad51 and incubated with Taxol for 72 hr. Cell viability was determined by MTT assay. **I.** GR overcomes the Rad51-induced γ-tubulin reduction. In addition, Rad51 is obviously reduced by GR overexpression. GFP-tagged GR and HA-tagged Rad51 were transfected to HEK 293 cells. After 72hr, protein levels were detected with anti-GFP and HA antibodies. **J.** Obvious reduction of Rad51 by cortisone treatment in GR transfected HEK 293 cells. GFP-tagged GR was transfected to HEK 293 cells and cortisone (5 μM) was treated. After 72hr, western blot was performed. **K.** GR expression was not altered by VHL. GFP-tagged GR and HA-tagged VHL were transfected to HEK 293 cells and cortisone (5 μM) was treated. After 72hr, western blot was performed. Actin was used as loading control.

Since elevated expression of γ-tubulin can overcome the Taxol-induced cell death [19, 26–30]. We first measured the γ-tubulin expression. As we expected, cortisol/cortisone obviously induced γ-tubulin in all of tested cell lines (Figure 2A and Supplementary Figure S2A), and FST did not block the γ-tubulin induction (Figure 2B). In this experiment, we also observed the reduction of BRCA1 in response to cortisol and cortisone (Figure 2B). Indeed, reduction of BRCA1 has been observed in Est-mediated γ-tubulin induced condition [19, 31, 32]. However, in ER-α negative cell lines, cortisone could induce γ-tubulin overexpression (Supplementary Figure S2B). In addition, they could promote MTOC amplification (Supplementary Figure S2C). Indeed, cortisone-treatment could increase the average number of mitotic MTOC from 2 to 3 (Figure 2C and 2D).

### Glucocorticoid receptor can promote MTOC amplification

Since cortisol and cortisone are glucocorticoid hormone and their signaling is mediated by glucocorticoid receptor (GR), we checked the involvement of GR on MTOC amplification. Transfection of GR alone could increase MTOC number, which results in outcomes similar to cortisone treatment (Figure 2E and 2F). In addition, GR could block the Taxol-induced cell death (Figure 2G). So, we next checked the effect of GR on Rad51-mediated Taxol sensitization. In our previous literature, Rad51 overexpression can re-sensitization in Taxol-resistant ER-α elevated cells and VHL deficient cell lines [19]. Interestingly, GR overexpression could block the Rad51-mediated Taxol re-sensitization (Figure 2H) via reduction of Rad51 (Figure 2I). Indeed, GR overexpression and cortisone treatment could reduce endogenous Rad51 expression (Figure 2J). However, differentially from ER- α, GR expression was not altered by VHL status. VHL overexpression or knock down did not alter the GR expression (Figure 2K and Supplementary Figure S2D), and FST did not make change in GR expression (Supplementary Figure S2E). These results indicate that stress hormone induced MTOC amplification and γ-tubulin elevation are achieved by GR as VHL independent mechanism.

### Favorable effect of GR inhibitor on stress hormone-mediated MTOC deregulation

**Figure 3:**
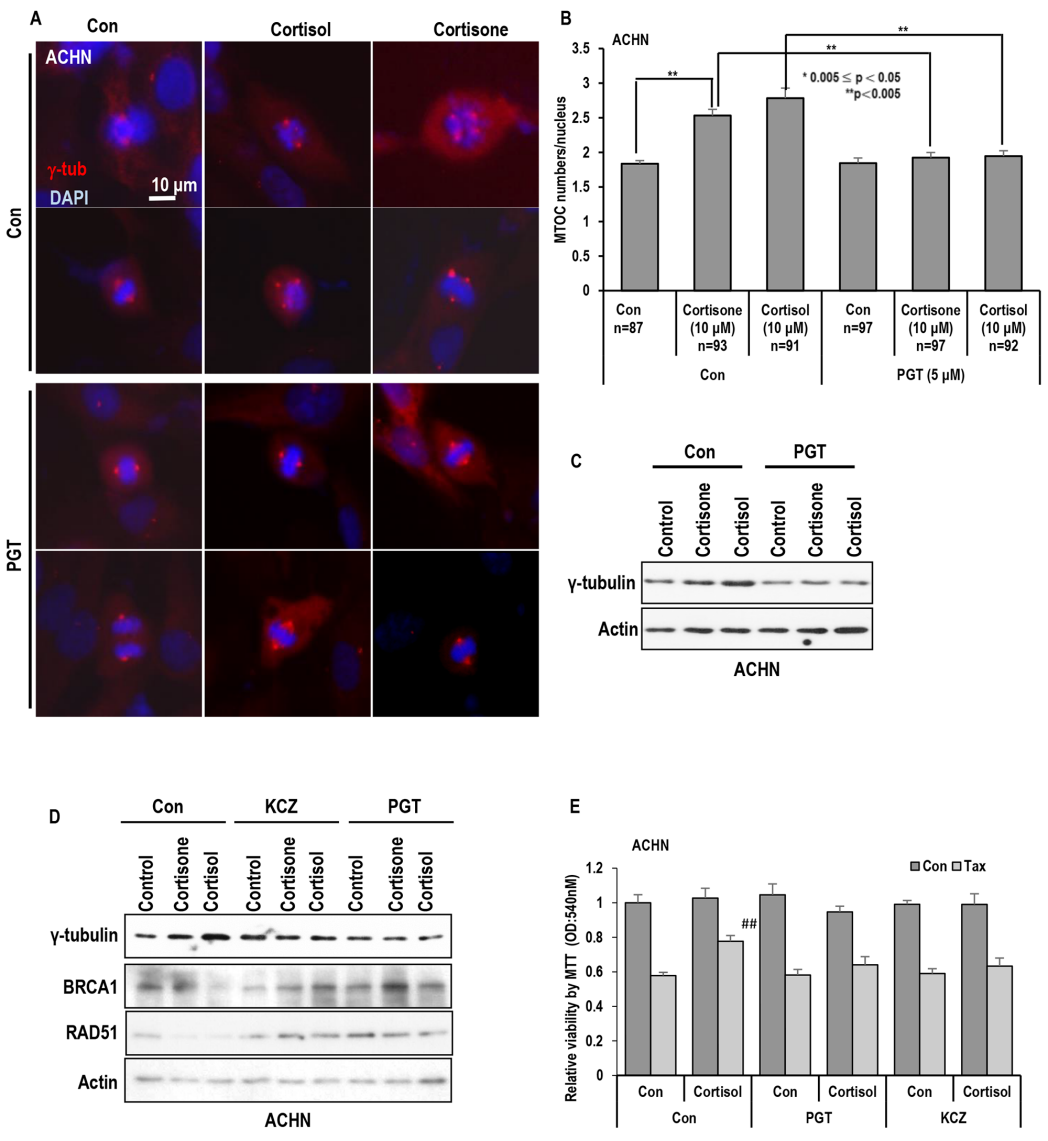
Inhibition of GR block the MTOC amplification **A.** and **B.** GR antagonist PGT (Progesterone) blocked the MTOC in ACHN cells. PGT (5 μM) treated cells were blocked MTOC amplication. About 80 cells were counted in each conditions to check average MTOC numbers B and representative pictures were shown A. Cells were stained with anti-γ-tubulin antibody (Red) to detect MTOC and DAPI (Blue) for DNA. **C.** Treatment of PGT abolished the stress hormone-induced γ-tubulin induction. Western blot analysis was performed with indicated antibodies. Actin was used as loading control. **D.** Other chemical antagonist of GR, Ketoconazole (KCZ) also blocked the stress hormone effects in ACHN cells. PGT (5 μM) and KCZ (5 μM) were treated. 72 hr. Each protein expression were determined by Western blot. Actin was used as loading control. **E.** GR antagonist overcome the cortisol induced Taxol-resistance. Cortisol (5 μM), and Taxol (3 μM) were treated for 72 hr in ACHN cells. PGT (5 μM) and KCZ (5 μM) were treated at the same time. Cell viability was measured by MTT assay. ## mean different group from other tested group by ANOVA test (p<0.001).

To confirm the involvement of GR on MTOC amplification, we tested the effect of progesterone (PGT) on stress hormone-induced MTOC amplification. PGT is well confirmed antagonist of GR [33–35]. Treatment of PGT abolished the stress hormone-induced MTOC amplification (Figures 3A, 3B and Supplementary Figure S3A) as well as γ-tubulin induction (Figure 3C). Since PGT is also hormone, we used other chemical antagonist of GR, ketoconazole (KCZ) [36, 37]. KCZ as well as PGT blocked the γ-tubulin induction and the reduction of Rad51 and BRCA1 (Figure 3D and Supplementary Figure S3B). Moreover, these inhibitors overcome the cortisol induced Taxol-resistance (Figure 3e).

### GR binds to Rad51 and disrupts BRCA1-Rad51 binding

**Figure 4:**
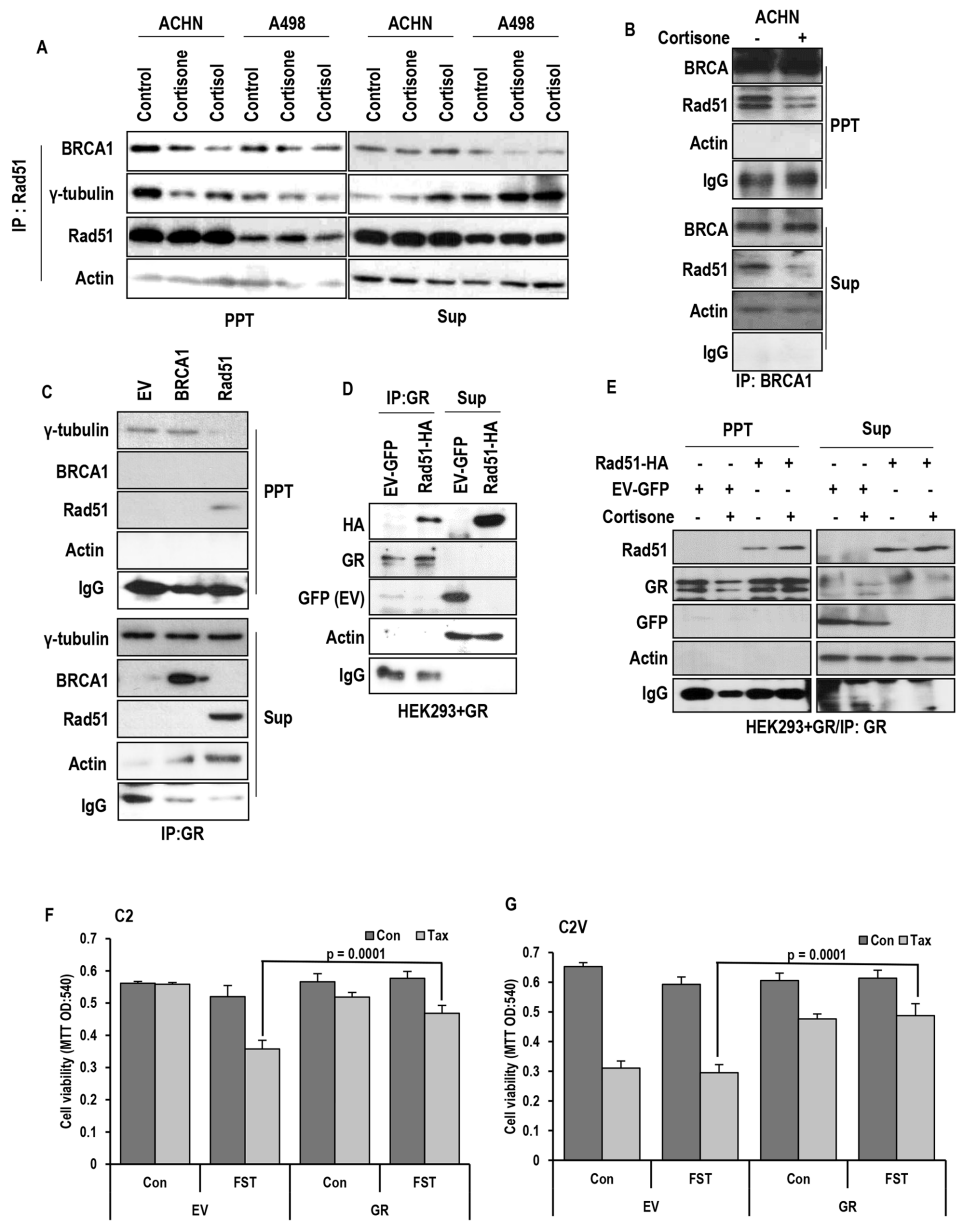
GR binds to Rad51 and disrupt Rad51-BRCA1 interaction **A.** Cortisone (5 μM) and cortisol (5 μM) disrupt binding Rad51-BRCA1 interaction in VHL-positive ACHN cells, but tiny effect on VHL-negative A498 cells. For binding assay, immunoprecipitation (IP) analysis was performed by anti-Rad51 antibody. **B.** Rad51-BRCA1 binding is reduced by cortisone treatment in VHL-intact ACHN cells. Anti-BRCA1 antibody was used for IP analysis. **C.** GR only binds to Rad51 but not BRCA1. **D.** The binding of GR-Rad51 is confirmed by exogenous proteins. GFP-tagged GR and HA-tagged Rad51 were overexpressed in HEK293 cells, and lysates from these cells were used for IP analysis. **E.** Enhanced binding of Rad51-GR is detected by cortisone (5 μM) treatment. IP analysis was performed by anti-GR antibody. **F.** Taxol sensitivity induced by FST in VHL-negative C2 cells is blocked through GR overexpression. **G.** Cortisone induced Taxol resistance is not affected by inhibition of ER-α in VHL-intact C2V cells, which has Taxol sensitivity. After transfection for GR overexpression, Taxol (Tax; 3 μM) and Fulvestant (FST; 3 μM) were treated as indicated. 72 hr later, MTT assay was performed for measuring cell viability.

Since Est/ER-a-induced MTOC amplification is achieved by dissociation of Rad51 and BRCA1 [19, 31, 32], we next checked the effect of glucocorticoid hormones (GH) on the interaction between Rad51 and BRCA1 or γ-tubulin. In RCC cell lines, we could observe the dissociation of BRAC1 and g-tubulin from Rad51 in response to GH (Figure 4A). The dissociation of BRCA1-Rad51 by cortisone was also confirmed by IP analysis with BRCA1 Ab (Figure 4B). Among BRCA1 and Rad51, which one is binding target of GR is next question. To address which one is target of GR between BRCA1 and Rad51, IP analysis was performed with GR. Differentially from ER-α that is evenly associated with both proteins, GR showed the binding affinity with only Rad51 (Figure 4C). The binding between Rad51 and GR was confirmed by exogenous proteins (Figure 4D). Although the binding of Rad51 and GR was detected without Cortisone, GH enhanced the binding (Figure 4E), perhaps, due to GR translocation into nucleus. To eliminate the involvement of ER-α in this reaction, GR-induced Taxol resistance was measured under FST-treated condition. Although FST could sensitize Taxol in VHL-deficient C2, GR could overcome the FST-induced sensitization (Figure 4F). Moreover, FST did not block the GR-induced Taxol resistance in VHL intact C2V (Figure 4G). These results indicate that GR/GH signaling can disrupt Rad51-BRCA1 binding via ER-α independent mechanism.

### Rare ginsenosides sensitize to taxol-induced cell death

**Figure 5:**
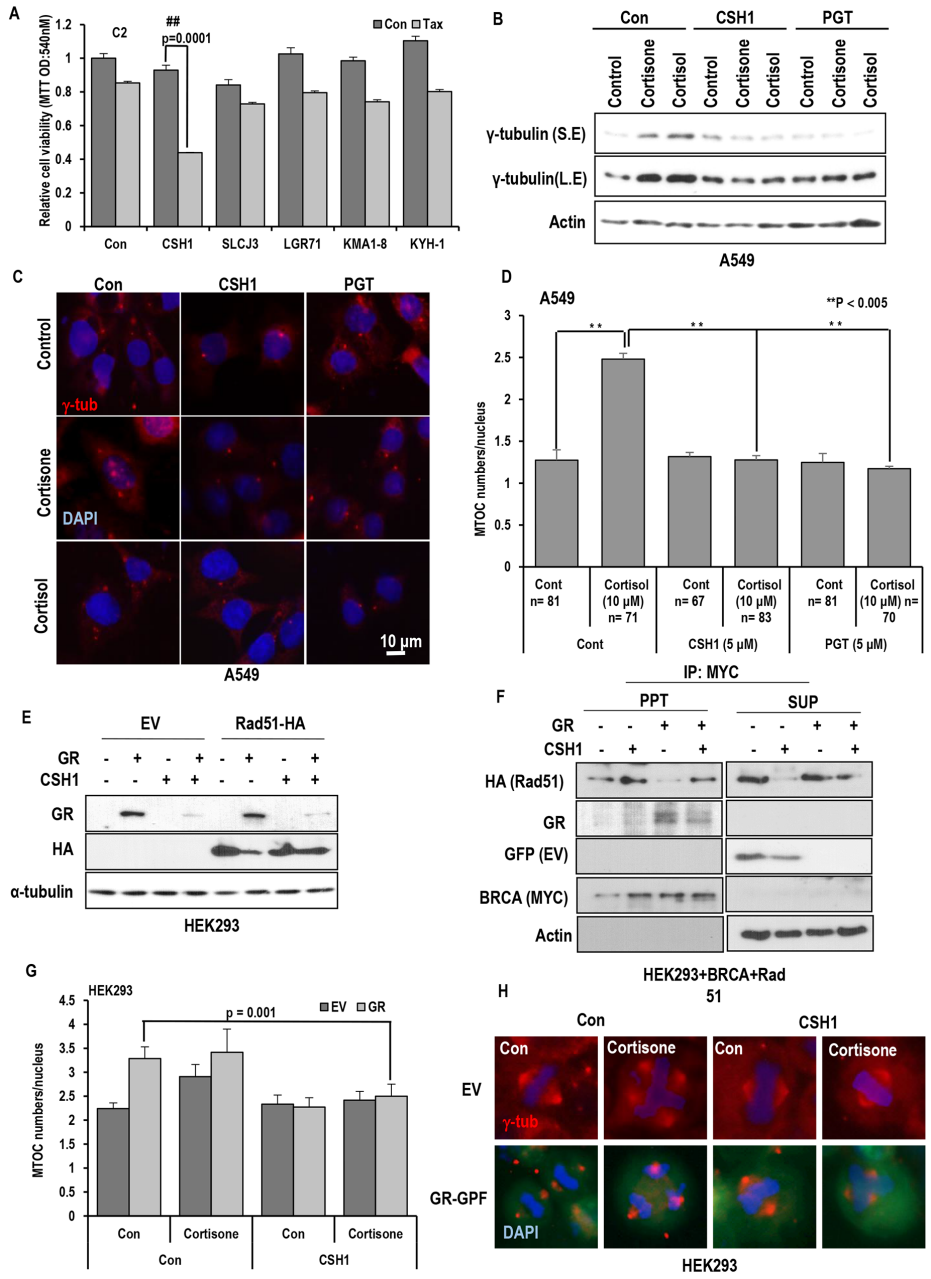
The effect of Rare ginsenoid on Taxol-induced cell death **A.** The effect of various ginsenosides on Taxol-induced cell death. Only CSH1 (Rg6) showed the Taxol sensitization effect in C2. Each ginsenosides (5 μM) and Tax (3 μM) were treated. After72 hr, cell viability was measured by MTT assay. **B-D.** PGT, CSH1 could abolish the stress hormone-induced γ-tubulin expression in A549 (Lung cancer cell line). PGT (5 μM), CSH1 (5 μM), Cortisol (5 μM) and Cortisone (5 μM) were treated for 72 hr. Western blot was performed for measuring γ-tubulin expression. Actin was used as loading control. Approximately 70 cells were counted for average MTOC numbers and representative pictures are shown. Cells were stained with anti-γ-tubulin antibody (Red) and DAPI (Blue). **E.** GR expression is suppressed by CSH1. GR induced suppression of Rad51 expression was recovered by CSH1 treatment. **F.** CSH1 could promote the interaction of BRCA1 and Rad51 and overcome the GR-disrupted binding of them. MYC-tagged BRCA1, HA-tagged Rad51 and GFP-tagged GR were overexpressed in HEK293 cells, and lysates from these cells were used for IP analysis for binding assay. IP analysis was performed by anti-MYC antibody. **G** and **H.** CSH1 could block the GR or cortisone induced MTOC amplification. Approximately 50 cells were counted for average MTOC numbers and representative pictures are shown. GFP-tagged GR was transfected to HEK 293 cells. CSH1 (5 μM) and cortisone (5 μM) were treated as indicated. Cells were stained with anti-γ-tubulin antibody (Red) and DAPI (Blue).

In our previous result, we showed the proper inhibition of stress hormone signaling could sensitize to Taxol as well as suppression of MTOC amplification (Figure 3). Thus, we looked for candidate chemicals that can suppress stress hormone-GR network. In oriental medicine, *Panax Ginseng* has been used for various kinds of human cancers and kidney diseases [21, 22]. In fact, very rare ginsenoside, CSH1 (RG6) possess very similar chemical structure with cortisone, cortisol or Est (Supplementary Figure S4A). So, we checked the effect of Rg6 on Taxol-induced cell death, comparing with other general ginsenosides. Interestingly, CSH1, but not other general ginsenosides, sensitized C2 cell to Taxol-induced cell death (Figure 5A). We could also observed the similar effect of CSH1 in A498 (Supplementary Figure S4B). To get more detail information about this, we evaluated the Taxol-sensitizing effect of CSH1-related ginsenosides (CSH2-4; Supplementary Figure S4C) and found that CSH3 also possessed the similar effect with CSH1 in Taxol-induced cell death, like as FST (Supplementary Figure S4D). However, in Taxol-sensitive C2V [19], these chemicals did not show additional sensitization to Taxol (Supplementary Figure S4D). So, we next checked the effect of CSH1 on γ-tubulin induction by cortisol and cortisone. Similarly to PGT, CSH1 could abolish the GH-induced γ-tubulin expression (Figure 5B) as well as MTOC amplification (Figure 5C and 5D) in human lung cancer cell line, A549. We could confirm the beneficial effect of CSH1 in human colon cancer cell line, HCT116 (Supplementary Figures S4E and S4F). These results indicate that CSH1 can suppress MTOC amplification in regardless of cell types.

### CSH1 blocks GH-induced taxol resistance and MTOC amplification

To get more detail biological effect of CSH1, we monitored the effect of CSH1 on Rad51 and GR expression. CSH1 could suppress GR expression and reduction of Rad51 by GR-overexpression (Figure 5E). In addition, CSH1 could promote the interaction of BRCA1 and Rad51 and overcome the GR-disrupted binding of them (Figure 5F). These results led us to check the number of MTOC. As we expected, increase of MTOC by cortisone or GR overexpression was completely abolished by CSH1 treatment (Figure 5G and 5H).

### CSH1 inhibits GR translocation and chromosome instability

**Figure 6:**
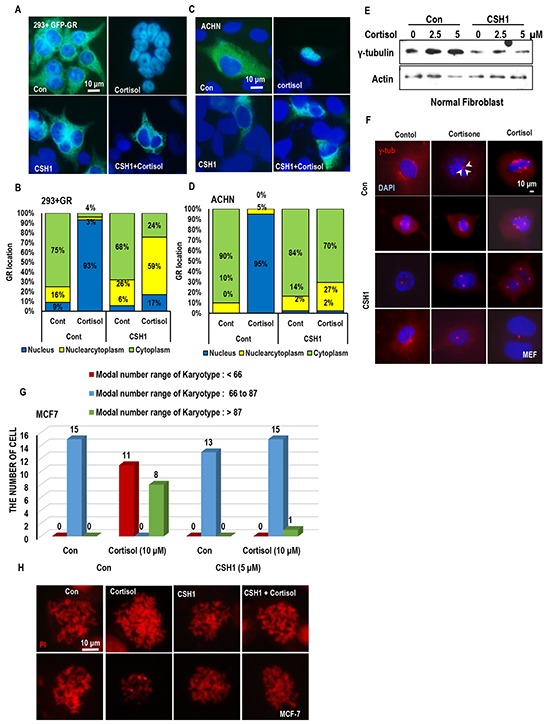
CSH1 blocks GR translocation and chromosomal abnormality **A** and **B.** CSH1 blocks GR-translocation in 293 cells. 293 cells were transfected with GFP-GR for 24 hr and incubated with cortisol and CSH1. Representative pictures were provided in A. based on the pictures, cell counting was performed. Nuclear GR (blue bar) in response to cortisol was abolished by CSH1. **C** and **D**. The same experiments with A and B were performed with ACHN and obtained the similar results. **E**. Cortisol induces g-tubulin in normal human fibroblast. Normal fibroblasts (obtained from 81 year-old) were incubated with cortisol for 24 hr. **F.** MTOC amplification in mouse embryonic fibroblast (MEF) is induced by cortisol and cortisone. MEF, obtained from 13.5 day, were incubated with 5 μM of indicated stress hormone for 72 hr. cells were fixed and stained with γ-tubulin Ab (red). DAPI was used for DNA staining. **G** and **H**. CSH1 blocks chromosomal abnormality. MCF-7 (normal chromatin number is 66 to 87) were incubated with stress hormones and CSH1 for 72 hr. Cells were arrested in M-phase by nocodazole and chromosomes were spread out. Number of chrmosomes were counted under fluorescence microscopy.

To know the working mode of CSH1, we checked the translocation of GR in CSH1-treated 293 cells. In GR-transfected 293, CSH1 blocked the cortisol-induced GR translocation into nucleus (Figure 6A and 6B). We could also observe the same effect of CSH1 in human RCC cell line (ACHN; Figure 6C and 6D) as well as other kinds of human cancer cell lines (A549, HCT116 and MCF-7; Supplementary Figure 5A-F). In all of tested cancer cell lines, CSH1 obviously blocked the translocation of GR in response to GH. So, our next question was that GH could induce MTOC amplification and CSH1 could block it. To address this, we measured the expression of γ-tubulin in human normal fibroblast after treatment of cortisol. Similarly with cancer cell lines, GH induced the γ-tubulin expression and CSH1 could block it (Figure 6E). In addition, cortisol and cortisone could induce MTOC amplification in normal mouse embryonic fibroblast (MEF) and CSH1 could suppress it (Figure 6F). Since MTOC amplification lead to chromosomal instability and aneuploidy that is one of important cause of cancer [15–18], we counted the chromosome numbers in GH-treated MCF-7 cells. In cortisol-treated cells, high or low number of chromosome-containing cells were obviously increased (Figure 6G and 6H). However, CSH1 blocked the GH-induced deregulation of chromosome numbers (Figure 6G and 6H). Since CSH1 can block the MTOC amplification, aneuploidy and γ-tubulin deregulation, we assumed that this chemical would be useful for cancer prevention. In addition, we wondered that continuous treatment of stress hormone can induce transformation. To address this, 2 kinds of normal human fibroblasts were cultured under low serum condition for 1 month with/without cortisol and CSH1. After 1 month, cells were seeded into 96 well plate and incubated for additional 2 weeks without serum. Under harsh condition, normal fibroblast obtained from 94 old person survived only in 2 wells (Supplementary Figures S6A and S6B). In contrast, large portion of cortisol-treated cells survived (Supplementary Figures S6C and D; 61/96 well, 85/96 well). At this condition, CSH1 co-treatment obviously reduced the survival rate (Supplementary Figures S5E and S5F). These results indicate that cortisol can promote the cell survival under unfavorable condition and inhibition of GR pathway by CSH can overcome the stress-hormone-induced improper cell survival.

### CSH1 shows the similar effect with FST

**Figure 7:**
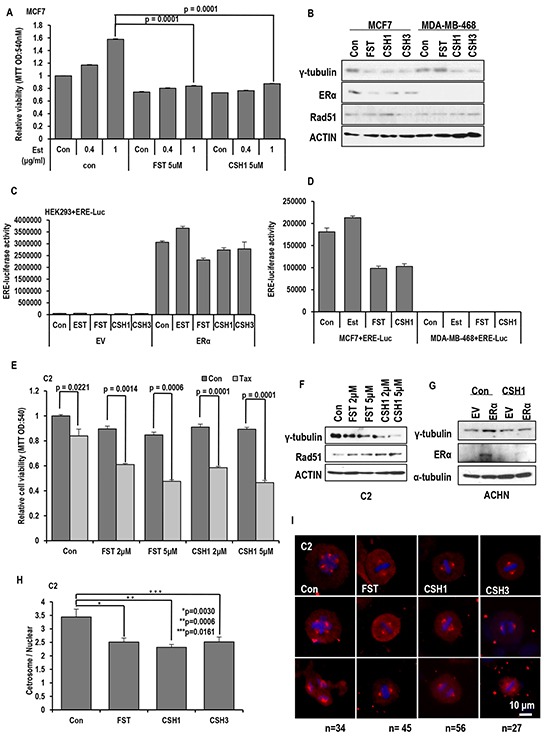
Similar effect of CSH1 with FST **A.** Inhibitory effect of CSH1 on Est-induced cell proliferation. Proliferation of ER-α positive MCF-7 cells was increased by Est-treatment in a dose-dependent manner. Similarly with FST, CSH1 suppresses the Est-induced proliferation in MCF-7. Chemicals were treated as indicated. 72 hr later, MTT assay were performed to check cell viability. **B.** Suppression of γ-tubulin expression by FST is only in MCF-7 cells, but CSH1 could reduce γ-tubulin in both MCF-7 and MDA-MB-468 cells. **C.** ER-α mediated transcription activity is reduced by CSH1 and CSH3 in ER-α transfected HEK293 cells. ERE-luciferase vectors and ER-α were co-transfected in HEK 293 cell to estimate ERE-luciferase activity. **D.** The similar result is reproduced in MCF-7 cells. CSH1 effectively suppressed ERE luciferase activity like FST. ERE-luciferase vectors were transfected to ERα-positive MCF7 cells and ERα-negative MDA-MB-468 cells. ERE- Luciferase assay was performed after the 4 hr treatment of each chemical. **E.** Taxol-induced cell death is provided by CSH1 similar to FST in VHL-negative C2 cells with dosage-dependent sensitization. **F.** Western blot analysis shows that expression of γ-tubulin and ER-α are decreased but Rad51 expression is increased by CSH1 treatment. **G.** The expression of ER-α is suppressed by CSH1. ER-α was transfected to VHL-positive ACHN cells. After the transfection, CSH1 (5 μM) was treated. 72 hr later, western blot was performed. **H** and **I.** The MTOC amplification is inhibited by rare ginsenosides compared with FST in VHL-negative C2 cells. About 50 cells were counted for average numbers of MTOC in each indicated conditions and representative pictures are shown. FST (5 μM), CSH1 (5 μM) and CSH3 (5 μM) were treated to VHL-negative C2 cells. After 72 hr, IF staining was performed. Cells were stained with anti-γ-tubulin antibody (Red) and DAPI (Blue).

Since CSH1 and FST could sensitize C2 to Taxol, the possibility that CSH1 can replace FST was tested. First, Est-induced cell proliferation was monitored. Similarly with FST, CSH1 could block the Est-induced cell proliferation in ER-α positive MCF-7 cell line (Figure 7A). In addition, CSH1 could mimic FST-induced γ-tubulin and ER-α suppression (Figure 7B). However, they did not alter the basal Rad51 expression (Figure 7B). ER- α-mediated transcription activity was also reduced by CSH1 in exogenous ER-α transfected 293 (Figure 7C). It was also confirmed in MCF-7 that ERE-Luc activity was reduced by CSH1 as strongly as FST (Figure 7D). These results indicate that CSH1 might serve as ER-α inhibitor. So, Taxol-sensitivity was also investigated. Similarly with dosage-dependent sensitization of FST, CSH1 also increased Taxol-induced cell death following dose dependent manner in C2 (Figure 7E). In addition, CSH1 could increase Rad51 expression in VHL-deficient C2 cell, like as FST (Figure 7F). Since FST can suppress ER-α expression [19, 39, 40], it was also tested. Exogenous ER-α was obviously reduced by CSH1 (Figure 7G), and CSH1 could show very resemble function to FST. Finally, we checked the CSH1 effect that can restore the MTOC, compared with FST [19]. CSH1 as well as CSH3 also restored the MTOC (Figure 7H and 7I). These results suggest that CSH1 and CSH3 could have the biological function as FST.

## DISCUSSION

In general, stress has been speculated for a long time as one of important cause of human cancer [1–4]. Indeed, several epidemiological studies support this hypothesis. However, it has never been clearly verified by molecular biological studies. In this study, stress hormone-induced centrosome instability has been investigated. From several experiments, it has been revealed that cortisol and cortisone could induce MTOC amplification (Figure 2C and 2D). Also, increase of γ-tubulin expression is achieved by GH that is not related with ER-α signaling (Figure 2B). Indeed, induction of γ-tubulin was observed in ER-α negative MDA-MB 468 (Supplementary Figure S2B) and male-originated lung cancer cell lines and colon cancer cell lines (Figure 2A). Concerning this, GR is deeply involved. Actually, GR-overexpression could promote MTOC amplification without triggering of GH. Considering these results and previous result that ER-α promotes MTOC amplification, GH and Est might share common MTOC amplification property. In fact, Rda51-BRCA1 binding is common target for ER-α and GR. Although this study show that ER- α/GR signaling promotes MTOC amplification through disruption of Rad51-BRCA1 binding, it is not clearly demonstrated how Rad51-BRCA1 regulate MTOC [19].

In this study, rare ginsenosides (CSH1 and CSH3) could block the Est/ER-α and GH/GR-mediated Taxol resistance and MTOC amplification. Indeed, Panax Ginseng as been widely its usage for several human diseases including kidney malfunction and cancer in oriental medicine [21, 22]. More important fact is that Panax Ginseng has not shown obvious side effect despite it has been used for thousand years in oriental medicine. However, its molecular working mechanism and target point has not been clearly demonstrated until now. This study show that CSH1 can suppress ER-α and GR and block the MTOC amplification and Taxol-resistance. Since MTOC amplification induces chromosomal instability [19, 42, 43], it would be one of reason for cancer development. Thus, CSH1 would be useful for prevention of human cancer. Although it is an incomplete model, we showed the preventive effect of CSH1 on improper cell growth (Supplementary Figure S6).

Indeed, this study show that CSH1 can replace FST, which is popular for treating breast cancers. CSH1 can block the EST/ER-α signaling as well as GH/GR signaling. Thus, it would be useful for cancer treatment. Since, the anti-cancer effect has not been verified in mouse model, it is not proper time to say that CSH1 can replace FST. However, CSH1 seems to possess enough potential for anti-cancer theraphy. Thus, further study for this chemical should be investigated.

**Figure 8:**
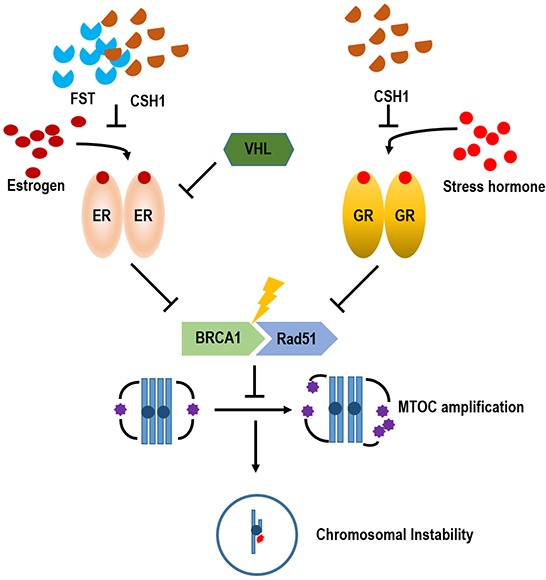
Summarized diagram Under stress conditions, GR pathway is activated, response to stress hormone and induces MTOC amplification and chromosomal instability. It would be one of putative tumorigenic mechanism for stress-induced cancer. However, unlike ER- α pathway, GR is not regulated by VHL. Since Rare ginsenoside, CSH1 possesses steroid-like backbone, it blocks GR as well as ER-α pathway-induced MTOC amplification and Taxol sensitivity. So, it would be used as anti-cancer drugs as well as cancer prevention strategy.

In summary, similarly with Est signaling, stress hormones also possess tumorigenic potential via MTOC amplification (Figure 8). It is also achieved by disruption of Rad51-BRCA1 binding. CSH1 can block the ability of ER-α and GR-mediated MTOC amplification (Figure 7). Thus, this chemical would be useful for anti-cancer drug for VHL-deficient RCC and Breast. For this, more intensive investigation should be performed.

## MATERIALS AND METHODS

### Cell lines and reagents

ACHN (VHL+) and A498 (VHL-) cells were purchased form Korea cell line bank. A549, HEK293, MCF-7 and MDA-MB-468 were obtained from American Type culture collection (ATCC, Manassas, VA). Other Cell lines (UMRC2; C2, UMRC2/VHL; C2V) were provided by Dr. Jung, YJ (Pusan National University). Human fibroblast cells were obtained from the Coriell Cell Repositories (New Jersey, USA). ACHN, A498, HEK293, MCF-7 and MDA-MB-468 were maintained in liquid DEME medium containing 10% FBS and 1% antibiotics at 37°C growth chamber. HCT116 cell lines were obtained from Dr. Vogelstein B (Johns Hopkins University). A549, HCT116 maintained in RPMI-1640 containing 10% FBS and antibiotics. Estrogen (250155), Fulvestrant (I4409), Taxol (T7402), Ketoconazole (UC280), Progesterone (P0130), Cortisone (C2755) and Cortisol (H4001) were purchased from Sigma (Missouri, USA). Antibodies against Actin (sc-1616), ER-α (sc-8002), GFP (sc-7392), and HA (sc-9996) were purchased from Santa Cruz (California, USA). Anti-γ-tubulin (T6557) was provided by Sigma (Missouri, USA), anti-glucocorticoid receptor (GR) Ab (12041) was obtained from Cell signaling (Massachusetts, USA). Rad51 (05–530), BRCA1 (07–434) were purchased from Milliopore (Darmstadt, Germany).

### Vectors and transfection

GFP-tagged GR (GR-GFP) and HA-tagged Rad51 (Rad51-HA) vectors were purchased from Addgene. The Myc-tagged wild type BRCA1 (BRCA1-Myc) was presented from Dr. Livingston, DM (Harvard Medical School). pVHL mammalian expression vectors were obtained from Dr. Jung, YJ (Pusan National University). Transfection was performed using Jetpei transfection agent (Polyplus New York, USA) for mammalian expression of these vectors. Briefly, the vector (1.5 μg) was mixed with 1.5 μl of Jetpei reagent in 150 mM NaCl solution. The mixture is incubated for 15 min at room temperature. After the incubation, the mixture was added to the cell. After 3 hours, the serum-free medium was replaced to 10% FBS contained medium.

### Western blot analysis and protein interaction studies

Proteins were extracted from cells with RIPA buffer (50 mM Tris-Cl, pH 7.5, 150 mM NaCl, 1% NP-40, 0.1% SDS and 10% sodium deoxycholate). Samples were applied to SDS-PAGE, and transferred onto a PVDF membrane. Blotted membranes were incubated with primary antibodies for 1 hr to overnight at 4°C and HRP-conjugate species-matched secondary antibodies for 1 hr at room temperature. Peroxidase activity was detected by chemi-luminescence with ECL kit (Intron, Seoul, Korea). For immunoprecipitation (IP) analysis, whole-cell lysates were incubated first with the proper antibodies for 4 hr at 4°C and then with protein A/G agarose beads (Invitrogen, California, USA) for 2 hr at 4°C. After centrifugation and washing with RIPA, the precipitated immune-complexes were subjected to SDS-PAGE and western blot analysis.

### Immunofluorescence staining

Cells were seeded on a cover glass and transfected with the indicated vectors or treated with the indicated chemicals. After fixing with Me-OH for 30 min, the cells were incubated with blocking buffer [PBS + anti-human-Ab (1:500)] for 1 hours. After washing with PBS, the cells were incubated with anti-γ-tubulin antibody in blocking buffer (1: 100 to 200) for 4 hr and subsequently with FITC-conjugated or Rhodamine-conjugated secondary antibodies in blocking buffer (1: 500) for 2 hr. The nucleus was stained by DAPI. After washing with PBS, cover glasses were mounted with mounting solution (Vector Laboratories, Cambridgeshire, UK). The immunofluorescence signal was detected through fluorescence microscopy (Zeiss, Jena, Germany).

### MTT assay

To measure the cell viability, cells were treated with the indicated chemicals for 4 days. For the MTT assay, cells were incubated with 0.5 mg/ml of MTT solution (Calbiochem, Darmstadt, Germany) for 4 hr at 37°C. After removing the excess solution, the precipitated materials were dissolved in 200 μl DMSO and quantified by measuring the absorbance at 540 nm.

### Luciferase assay

To estimate ER-α promoter activity, ERE-Luc vectors were transfected into cells for 24 hr, and cells were treated with the indicated chemicals. After washing with wash buffer (Promega, Wisconsin, USA), the cells were lysed by lysis buffer (Promega, Wisconsin, USA). The luciferase activity was determined by a luminometer (MicroDigital, Gyeonggi-do, South Korea).

### Statistical analysis

All results are expressed as mean + s.e.m. and performed at least n=4 per group. To obtain the statistical significance, the Student's t-test was performed. For ANOVA test, Numeric variables were summarized by their mean±SD (standard deviation). Before Analysis of Variance (ANOVA) for group comparison is performed, we first applied Shapiro–Wilk test and Levene's test to check data normality and to assess the equality of variances, respectively. When both normality and homoscedasticity are met, one-way analysis of variance (ANOVA) was conducted to compare the difference of a response variable (difference; Control-Taxol) for each condition, and a Tukey's HSD test was performed for post-hoc multiple comparison. When the data are satisfied with neither normality nor homoscedasticity, a non-parametric method Kruskal-Wallis test was used to assess equality of multiple group means, and then Mann-Whitney U test was performed for post-hoc multiple comparisons. For adjusting family-wise Type I error rate, we applied Bonferroni correction to adjust the p-values of multiple testing. SPSS 21.0 was used for all statistical analysis.

## SUPPLEMENTARY FIGURES


