# Supplementary material for: Prevention effect of rare ginsenosides against stress-hormone induced MTOC amplification
Source: Oncotarget. 2016 Apr 27;7(23):35144–58. doi: 10.18632/oncotarget.9059 (PMC5085216; doi:10.18632/oncotarget.9059)
Supplement: Supplementary file 1 [file oncotarget-07-35144-s001.pdf]

SUPPLEMENTARY FIGURES

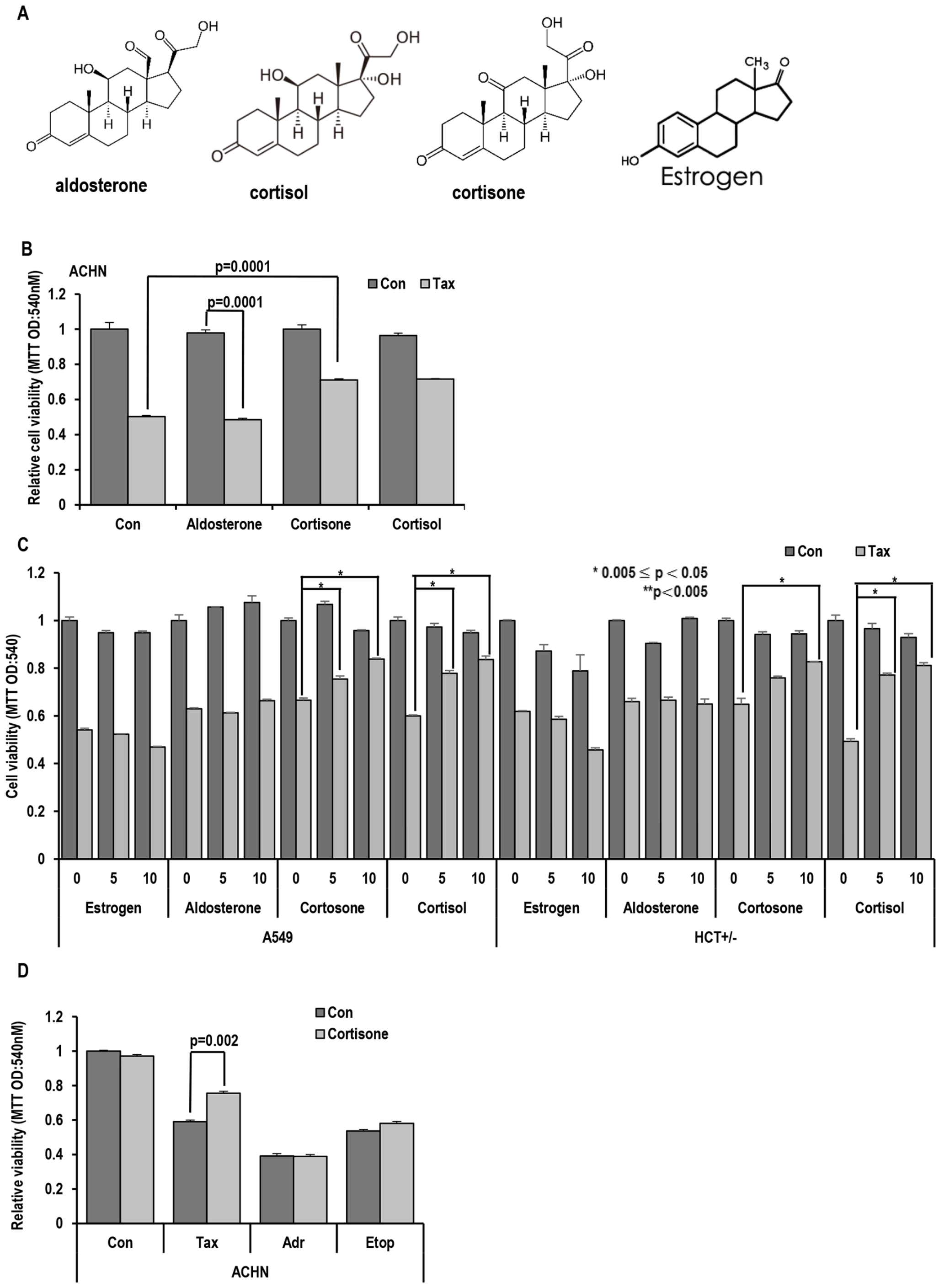

Supplementary Figure S1: Stress hormone induces Taxol resistance A. Chemical structures of glucocorticoid hormones (aldosterone, cortisol and cortisone) and estrogen. Since they are originated from cholesterol, they show very similar structure. **B.** ACHN shows the similar response with C2V. ACHN cells were incubated with the same condition of C2V. Cell viability was measured by MTT assay with 540 nm absorbance. **C.** Cortisol and cortisone but not aldosterone inhibited Taxol-induced cell death as dose-dependent manner in non-RCC cell lines. Aldosterone, cortisone, cortisol, and Taxol (3 μM) were treated for 72 hr in non-RCC lines. Cell viability was measured by MTT assay. **D.** The specific effect of cortisone on Taxol-induced cell death. Differentially from Taxol, cortisone did not provide the resistance to DNA damage reagent such as Adriamycin (Adr) and Etoposide (Etop). Cells were incubated with indicated chemicals (Taxol 3 μM; cortisone 5 μM; Adr 2 μg/ml; Etop 10 μM) for 72 hr later. Cell viability was determined by MTT assay.

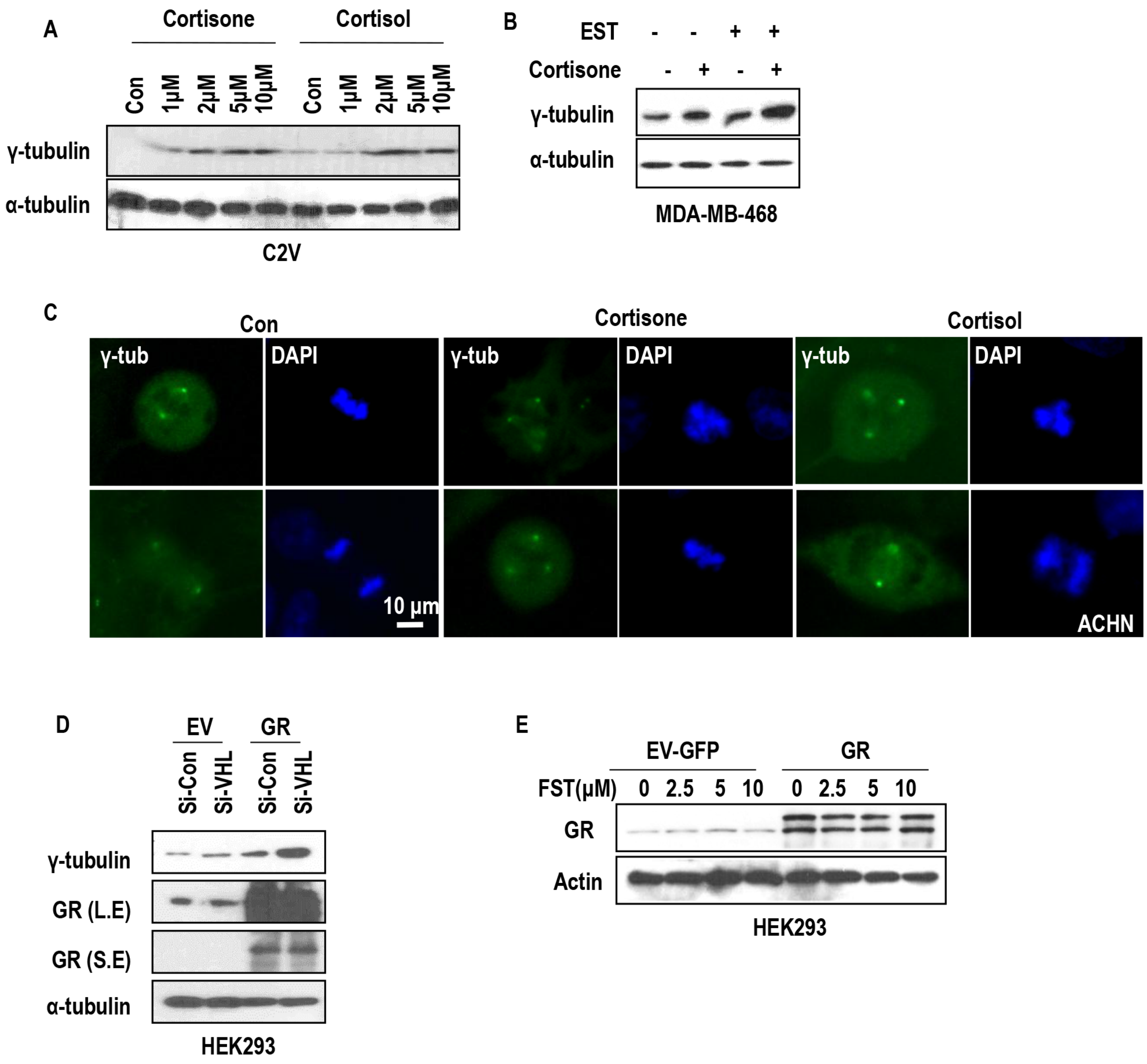

Supplementary Figure S2: Stress hormone increases MTOC via GR. A. The expression of γ-tubulin is increased by cortisone and cortisol in VHL-positive C2V cells, as dose-dependent manner. Protein expression was analyzed by western blot after 48hr of chemical treatment **B.** Induction of γ-tubulin expression in response to cortisone in ER-α negative MDA-MB-468 cells. The cells were incubated with Est (1 µg/ml) and cortisone (5 µM) for 72 hr. **C.** Stress hormone induce γ-tubulin in regardless of FST. A498 (VHL deficient cell line) were incubated with cortisone (5 µM) and cortisol (5 µM) with/without FST (3 µM) for 24 hr. However, BRCA1 expression was increased by FST, although reduction of BRCA1 expression by GH was not affected. In addition, p53 expression was not altered by FST or GH. Actin was used for loading control. **D.** VHL knock down did not alter the GR expression. Si-VHL and GFP-tagged GR were transfected for 24 hr in HEK293 cells. Western blot analysis was performed with indicated antibodies, and Actin was used as loading control. E. FST did not make change in GR expression. GFP-tagged GR were overexpressed in HEK293 cells. The cells were incubated with FST for 72 hr. GR protein expression were determined by western blot. Actin was used as loading control.

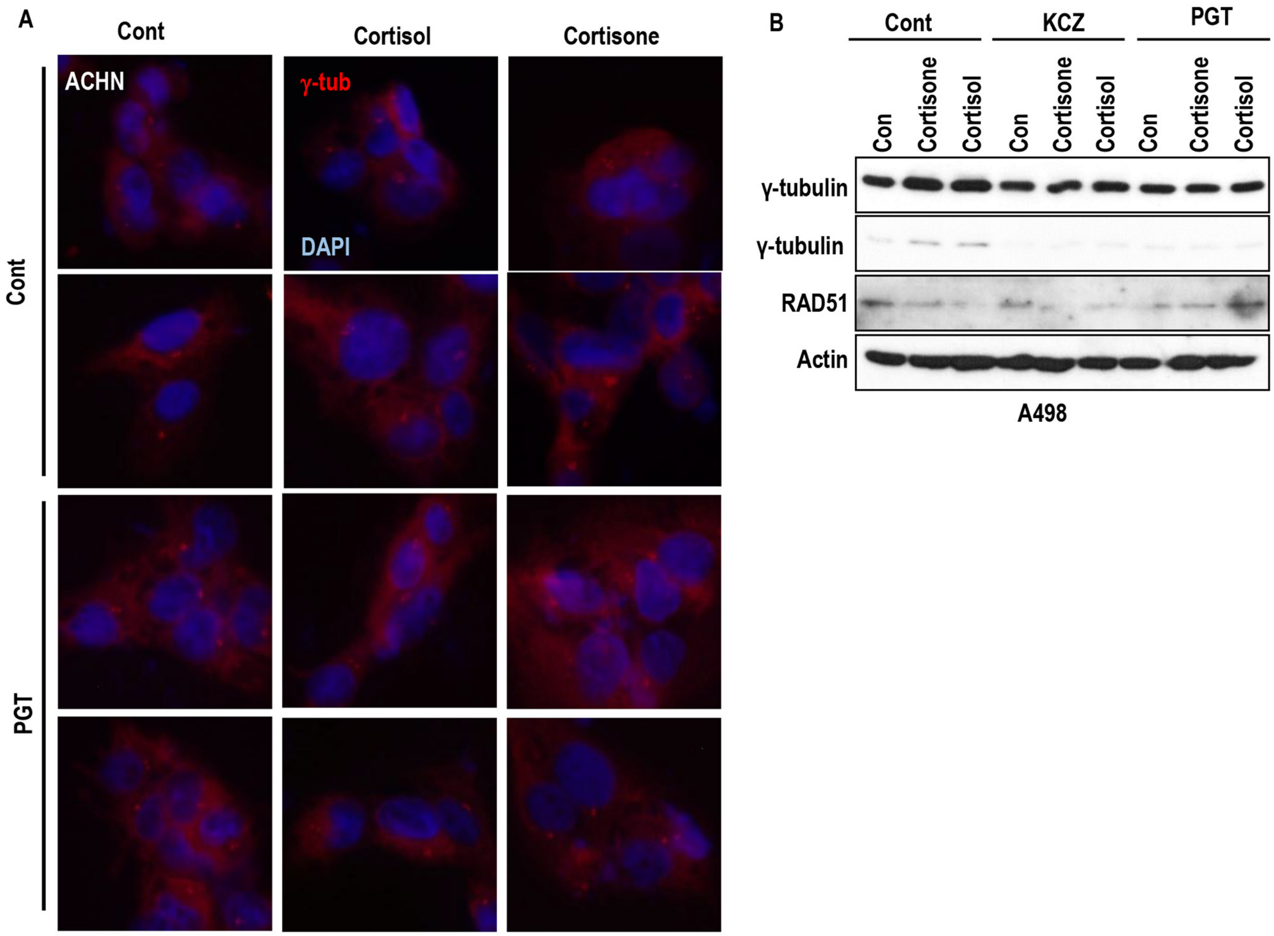

Supplementary Figure S3: Inhibition of GR block the MTOC amplification. A. GR antagonist PGT (Progesterone) blocked the MTOC in ACHN cells. PGT (5 μM) treated cells were blocked MTOC amplication. Cells were stained with anti-γ-tubulin antibody (Red) to detect MTOC and DAPI (Blue) for DNA. **B.** Treatment of PGT and KCZ abolished the stress hormone-induced effects in A498 cells. PGT (5 μM) and KZC (5 μM) were treated. 72 hr. Each protein expression were determined by Western blot. Actin was used as loading control.

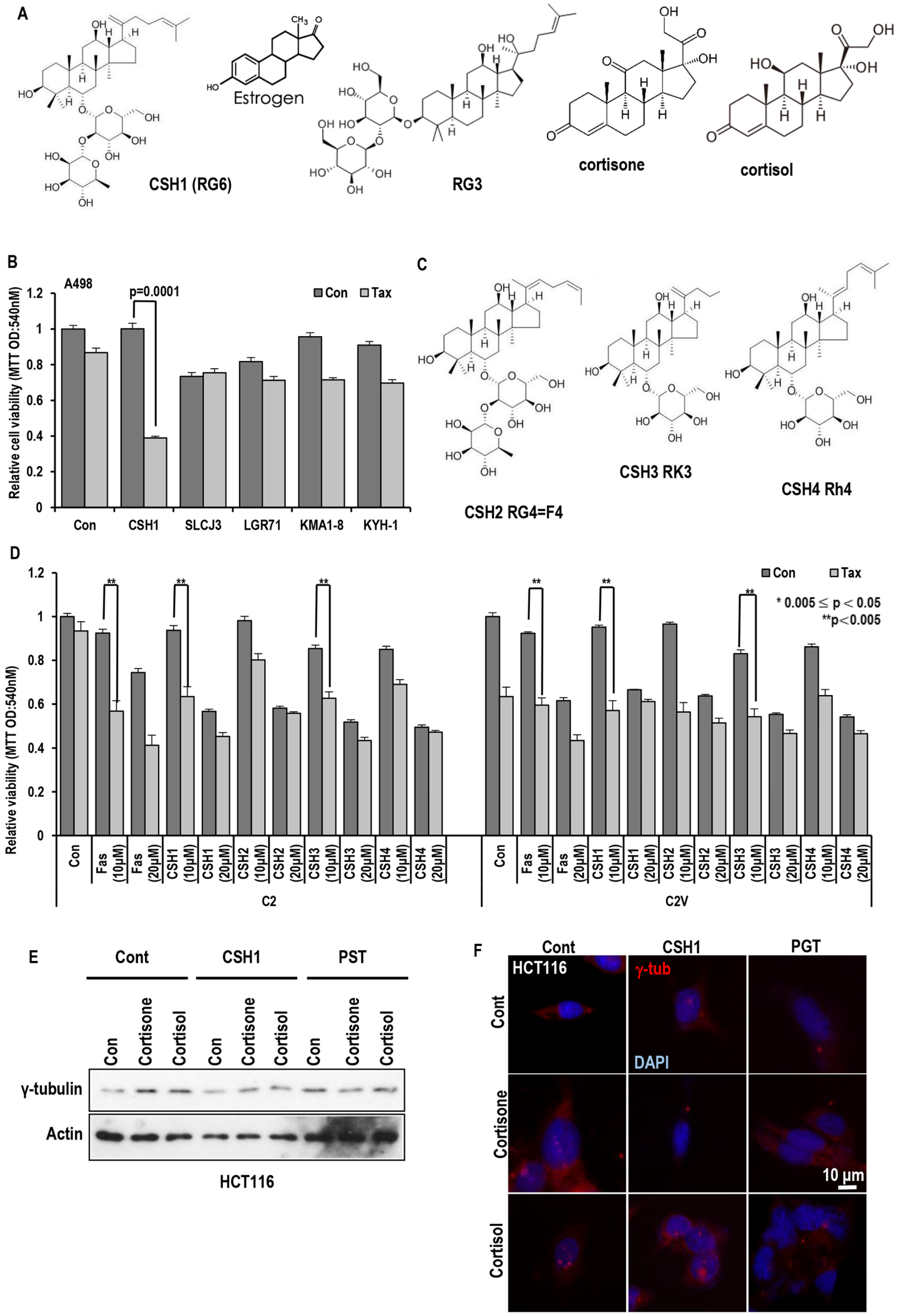

Supplementary Figure S4: Rare ginsenosides sensitize to Taxol-induced cell death. **A.** The chemical structures of Rare ginsenosides. Differentially from common ginsenoside (Rg3), sugars of CSH1-7-39-1 Rg6 (CSH1) are linked to second ring. However, they possesses the similar steroid structure with cortisone and estrogen. **B.** The effect of various ginsenosides on Taxol-induced cell death. Only CSH1 (Rg6) showed the Taxol sensitization effect in A498. Each ginsenosides (5 μM) and Tax (3 μM) were treated. After72 hr, cell viability was measured by MTT assay. **C.** The chemical structures of CSH1-resembled other ginsenosides (CSH2, CSH3 and CSH4). **D.** Taxol sensitivity is induced by CSH1 and CSH3 like FST in VHL-negative C2 cells which has Taxol resistance. Taxol (3 μM) and indicated concentration of Ginsenosides and FST were treated. After the 72hr, MTT assay was performed to check cell viability. **E.** and **F.** PGT, CSH1 could abolish the stress hormone-induced γ-tubulin expression in HCT116 (Colon cancer cell line). PGT (5 μM), CSH1 (5 μM), Cortisol (5 μM) and Cortisone (5 μM) were treated for 72 hr. Western blot was performed for measuring γ-tubulin expression. Actin was used as loading control. Cells were stained with anti-γ-tubulin antibody (Red) and DAPI (Blue).

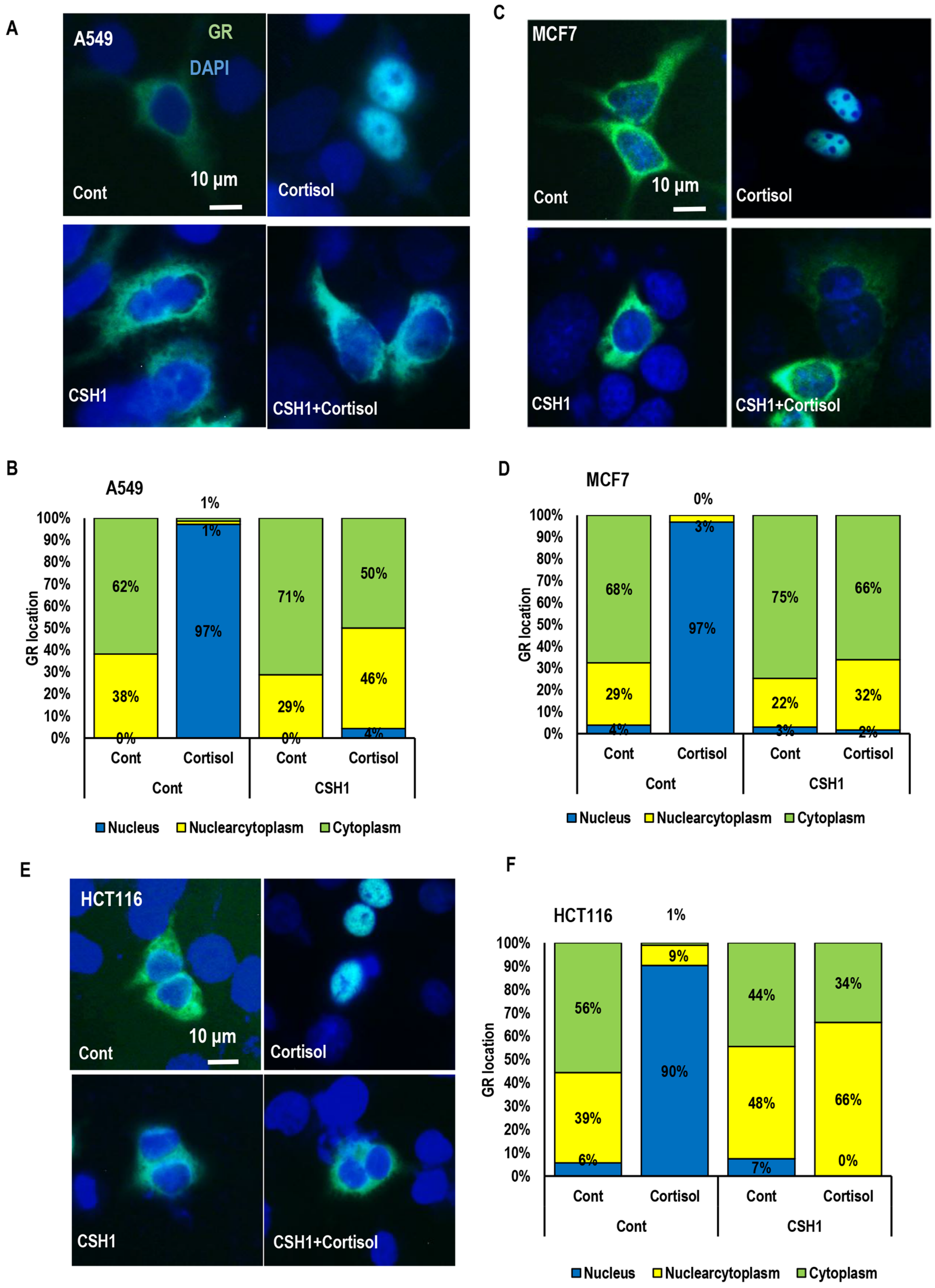

Supplementary Figure S5: CSH1 blocks GR translocation. In human cancer cell lines (A549, MCF-7 and HCT116), translocation of GR in response to Cortisol was obviously inhibited by CSH1. A549 (**A.** and **B.**), MCF-7 (**C.** and **D.**), and HCT116 (**E.** and **F.**) were incubated with cortisol and/or CSH1 for 24 hr. representative Figures were provided in A, C, and E. cell counting results based on GR-localization were also provided as bar-graph (B, D and F; N>50).

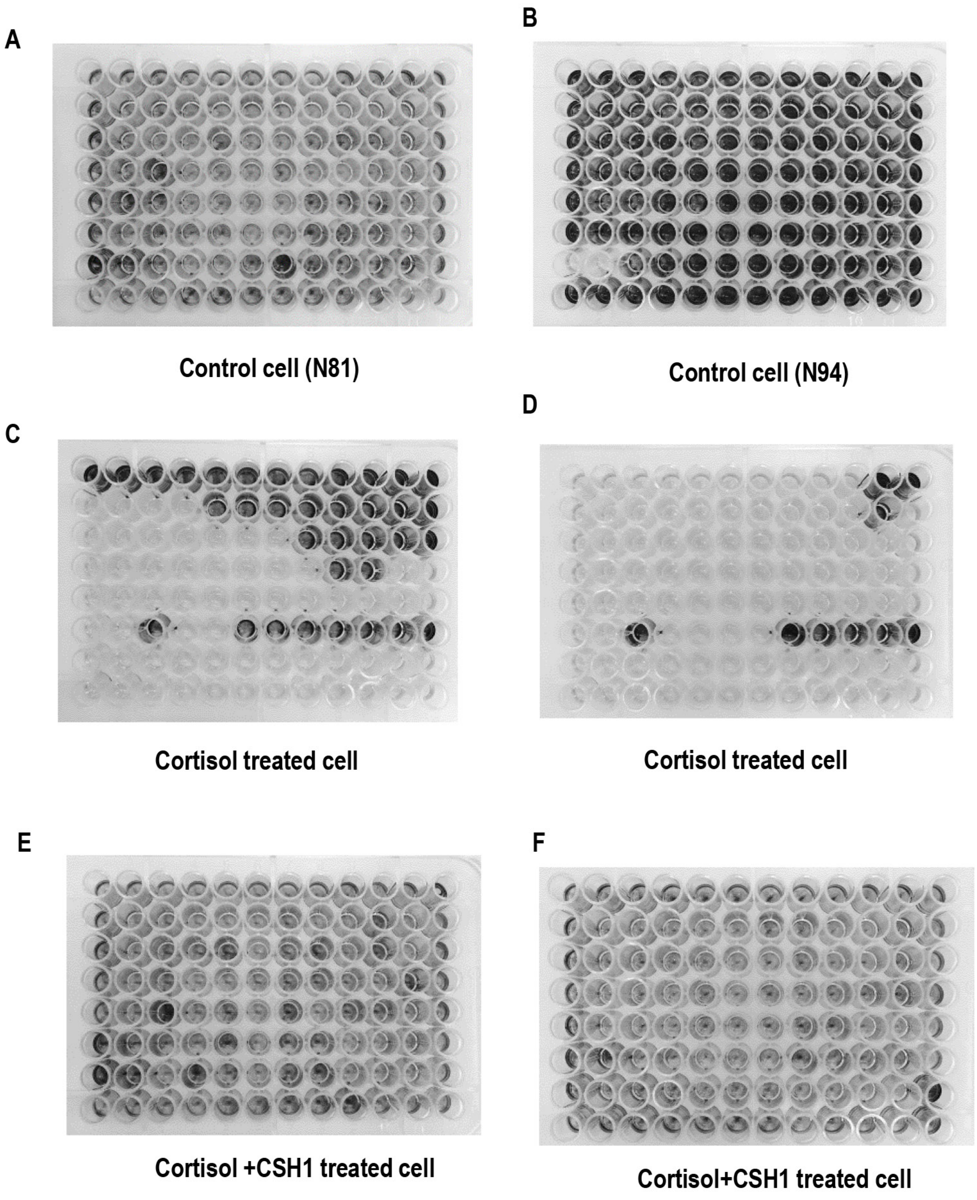

Supplementary Figure S6: CSH1 blocks stress hormone-induced mal-functions. A-D. Continuous treatment of stress hormone can induce transformation. 2 kinds of normal human fibroblasts were cultured under low serum condition for 1 month with/without cortisol and CSH1. After 1 month, cells were seeded into 96 well plate and incubated for additional 2 weeks without serum. Cell viability was measured by tryphan blue exclusion assy. (live cells; unstained, dead cells; blue)
